# Gut microbiomes of mobile predators vary with landscape context and species identity

**DOI:** 10.1002/ece3.3390

**Published:** 2017-09-12

**Authors:** Julia Tiede, Christoph Scherber, James Mutschler, Katherine D. McMahon, Claudio Gratton

**Affiliations:** ^1^ Institute of Landscape Ecology University of Muenster Muenster Germany; ^2^ Department of Crop Sciences University of Goettingen Goettingen Germany; ^3^ Department of Entomology University of Wisconsin‐Madison Madison WI USA; ^4^ Departments of Civil and Environmental Engineering and Bacteriology University of Wisconsin‐Madison Madison WI USA

**Keywords:** body condition, diet, exotic species, gut bacteria, insect–microbe interactions, insects, lady beetles, natural enemies

## Abstract

Landscape context affects predator–prey interactions and predator diet composition, yet little is known about landscape effects on insect gut microbiomes, a determinant of physiology and condition. Here, we combine laboratory and field experiments to examine the effects of landscape context on the gut bacterial community and body condition of predatory insects. Under laboratory conditions, we found that prey diversity increased bacterial richness in insect guts. In the field, we studied the performance and gut microbiota of six predatory insect species along a landscape complexity gradient in two local habitat types (soybean fields vs. prairie). Insects from soy fields had richer gut bacteria and lower fat content than those from prairies, suggesting better feeding conditions in prairies. Species origin mediated landscape context effects, suggesting differences in foraging of exotic and native predators on a landscape scale. Overall, our study highlights complex interactions among gut microbiota, predator identity, and landscape context.

## 1 INTRODUCTION

Animal guts harbor a vast diversity of microbes, as revealed by modern DNA‐based methods (Bahrndorff, Alemu, Alemneh, & Lund Nielsen, 2016; Engel & Moran, 2013; Gibson & Hunter, 2010). The gut microbiome may affect host fitness in many ways including host nutrition, regulating growth rate and stress tolerance, through protection against natural enemies, or by mediating host–pathogen interactions (Dillon & Dillon, 2004; Douglas, 2009; Ferrari, Darby, Daniell, Godfray, & Douglas, 2004; Henry, Maiden, Ferrari, & Godfray, 2015; Ruokolainen, Ikonen, Makkonen, & Hanski, 2016). Gut microbes can be vertically transmitted or acquired from the environment (horizontal transmission; Gibson & Hunter, 2010; Mason & Raffa, 2014). In addition, the total gut community also includes transient species that cannot permanently colonize the gut (Dillon, Vennard, Buckling, & Charnley, 2005; Erkosar & Leulier, 2014) but may represent a supplementary food source, or contribute to digestion (Bouchon, Zimmer, & Dittmer, 2016). Understanding factors influencing animal gut microbiome composition can thus yield important insights into ecological interactions.

Laboratory studies have found that the gut microbial community of many arthropod species is affected by host diet (Broderick, Raffa, Goodman, & Handelsman, 2004; Lundgren & Lehman, 2010; Mason & Raffa, 2014; Wang, Jin, & Zhang, 2011), either through effects of food substrates on the persistence of specific microbes, or directly from the acquisition of associated microbes (Bili et al., 2016; Chandler, Lang, Bhatnagar, Eisen, & Kopp, 2011). In addition, gut microbiota of wild insect populations vary geographically, suggesting that differences in the local environment can shape microbial assemblages (Adams, Currie, Gillette, & Raffa, 2010; Coon, Brown, & Strand, 2016; Toju & Fukatsu, 2011; Yun et al., 2014). The gut microbiome of wild insect populations likely represents a sample of microbiota from local food and other sources in their surrounding environment (Borer, Kinkel, May, & Seabloom, 2013). On a local scale (small quadrats of 0.025 m²), correlations among gut microbial richness of two ground‐dwelling cricket species and prey richness in the habitat have been reported (Schmid, Lehman, Brözel, & Lundgren, 2015); yet, the landscape‐level consequences for mobile organisms such as flying predators have remained largely unexplored.

Predator–prey interactions have frequently been shown to be influenced by landscape composition and structure. A multitude of studies has investigated numerical responses of predators to the surrounding landscape (Chaplin‐Kramer, O'Rourke, Blitzer, & Kremen, 2011; Gardiner et al., 2009; Liere et al., 2015), including predator movement (Blitzer et al., 2012; Forbes & Gratton, 2011; Schellhorn, Bianchi, & Hsu, 2014). If predators use multiple prey items located in different habitat types, landscape complexity should be positively correlated with diet items consumed (Bianchi, Schellhorn, & Cunningham, 2013; Bianchi, Schellhorn, & van der Werf, 2009; Layman, Quattrochi, Peyer, & Allgeier, 2007; Tscharntke, Klein, Kruess, Steffan‐Dewenter, & Thies, 2005), resulting in a greater variety of food‐related or environmental microbes in the predators′ guts. Yet, systematic studies on the effects of landscape context on predator gut microbiota are lacking.

Ideally, studies investigating landscape configuration and composition are performed in experimental landscapes, where landscape attributes are controlled by the experimenter (Hadley & Betts, 2016, p. 59). However, such studies are often performed within only a single habitat type and cover often cover less than 1 km² (Haddad et al., 2015); such scales are considerably smaller than the foraging range of many insects, including pollinators or predatory beetles. Here, we report results from a mensurative experiment, in which study sites are selected a priori on a meaningful biological scale. We present evidence for landscape‐level effects on insect gut microbiota on a scale of several thousand km².

Predator fitness may be affected by landscape context directly through variability in food quality and quantity. Prior work has shown that landscape context is associated with fitness‐related measures of body condition, such as body size or fat content, in ground‐dwelling predators (Bommarco, 1998; Öberg, 2009; Östman, Ekbom, Bengtsson, & Weibull, 2001), but this relationship has not been examined in mobile arthropod predators and the role of gut microbes has remained elusive. As the microbiome can directly affect the nutritional state and health of an organism (Bahrndorff et al., 2016; Borer et al., 2013; Gibson & Hunter, 2010; Ruokolainen et al., 2016), changes in the microbiome associated with the landscape could also have indirect microbe‐mediated effects on body condition.

In this study, we examined the effects of landscape context on the gut bacterial community and body condition of predatory insects. We used aphidophagous lady beetles as our study system, as they are locally widespread and important natural enemies of aphids in agricultural crops (Obrycki, Harwood, Kring, & O'Neil, 2009; Snyder, 2009) and seminatural habitats (Bianchi et al., 2013). Although aphids are their preferred prey, the lady beetles' food spectrum includes a broad range of other soft‐bodied arthropods, as well as fungal or plant resources (Dixon, 2000; Evans, 2009; Hodek & Honěk, 1996; Trilitsch, 1999; Weber & Lundgren, 2009). In a proof‐of‐concept laboratory experiment, we first show that even a single meal can increase the richness and alter the community composition of gut bacteria in individual beetles, indicating that diet diversity can affect gut communities. In a mensurative field experiment (Hadley & Betts, 2016), we sampled six lady beetle species that differ in their phylogenetic relatedness (including three in the same genus), origin (native and exotic), and body size to explore the contribution of host‐specific factors to differences in the gut microbiome and physiological response to landscape context. We tested the effects of landscape context at two spatial scales by sampling beetles in two field types with contrasting plant diversity: (1) species‐rich prairies and soybean monocultures that (2) were systematically selected to be surrounded by landscapes ranging from low to high proportion of land covered by annual crops in southern Wisconsin, USA. We expected that mobile predators that forage in prairies have access to a broader range of prey types compared to beetles foraging in soybean and therefore would have a richer gut community. Because mobile predators may forage on a landscape scale, we further predicted that lady beetles would have a relatively simpler gut community when the collection sites are surrounded by crop‐dominated landscape compared to sites surrounded by more natural habitats. In addition, we examined whether landscape‐mediated changes in predator gut microbiota were associated with differences in body condition, assessed using estimates of beetle fat content. Fat content reflects the available energy reserves for survival and reproduction and resistance to nutritional stress (Arrese & Soulages, 2010; Roma, Bueno, & Camargo‐Mathias, 2010). We predicted that prairies and landscapes with low proportions of arable land would foster greater body condition. We show that changes at the field and landscape scale affected the gut bacterial community and physiological response of predators, but the direction of the effect differed significantly between exotic and native species, raising the possibility of inherent differences in habitat use and foraging preferences among these groups.

## 2 MATERIAL AND METHODS

### 2.1 Feeding experiment

**Figure 1:**
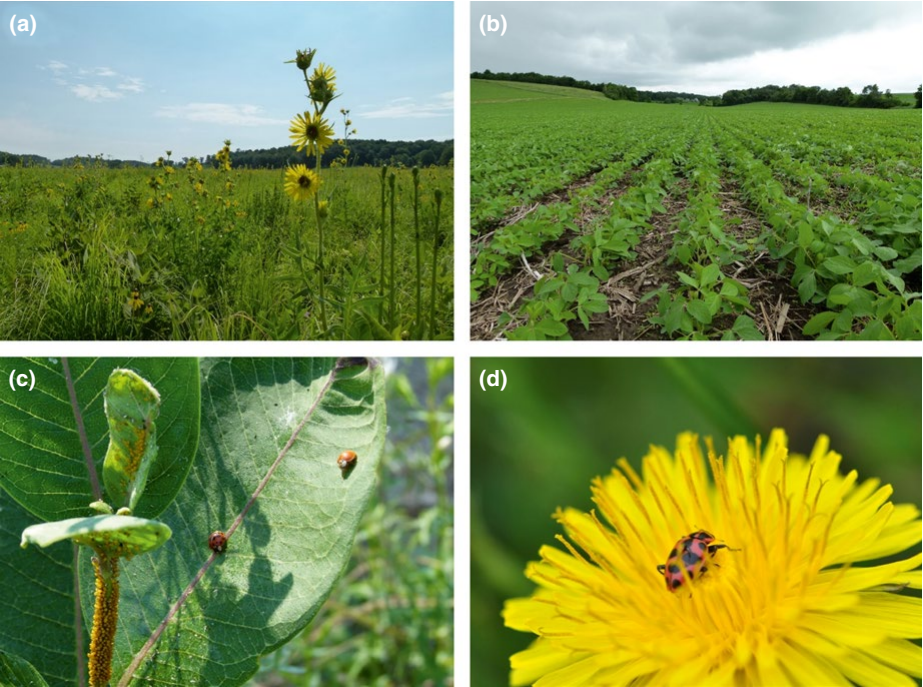
Examples for field study sites. (a) Restored prairie; (b) soybean field; (d) *Harmonia axyridis* on aphid‐infested milkweed (*Asclepias syriaca* L.) in a prairie (photo by J. Dreyer); (c) *Coleomegilla maculata* on dandelion (*Taraxacum officinale* L.)

In a laboratory feeding experiment, we tested whether a single meal has the potential to alter the gut bacterial community of lady beetles. Adult *Coleomegilla maculata* De Greer (pink spotted lady beetle) were collected in April 2012 in Arlington, Wisconsin (USA), from dandelion flowers where they commonly aggregate in the spring (Harmon, Ives, Losey, Olson, & Rauwald, 2000; Figure 1d). Beetles were maintained in the laboratory on dandelion flowers and moistened cotton balls for 7 days to allow their gut bacteria to equilibrate to similar diet environments. Prior to testing, beetles were starved for 48 hr. The beetles were randomly allocated to three treatments: (1) no food (control), (2) a meal consisting of one individual of *Acyrthosiphon pisum* Harris (pea aphid), and (3) a meal consisting of five different prey species (one individual each of *A. pisum, Rhopalosiphum padi* L. (bird cherry‐oat aphid), *Aphis gossypii* Glover (cotton aphid), and *Aphis glycines* Matsamura (soybean aphid)*,* and three eggs of *Spodoptera frugiperda* JE Smith ([Lepidoptera], beet armyworm). These species represent common prey of lady beetles in Wisconsin and the Midwestern USA. Beetles that finished their meal completely within 1 hr (*n* = 19 beetles) were transferred into 1.5‐ml microtubes containing 70% ethanol and frozen at −20 °C (*n* = 7 for the control, *n* = 5 for the 1‐species diet, and *n* = 7 for the 5‐species diet).

### 2.2 Field study

We sampled wild populations of lady beetles in southern Wisconsin, USA, in 2012. The region is dominated by agricultural row crops (mainly corn [*Zea mays* L.] and soybean [*Glycine max* L.]) with remaining patches of seminatural habitat (i.e., forest, grasslands, wetlands). We initially selected 10 prairies and 10 conventionally managed soy fields as two field types with contrasting diversity of plants and likely associated prey species. The fields were at least 2.6 km apart (Fig. S1 in Appendix S1). We analyzed the landscape composition within a 2 km radius of each field, which is an ecological meaningful distance for foraging flights in lady beetles (Woltz & Landis, 2014). The proportions of land cover types within each sector were analyzed with ArcGIS (10.0, ESRI, Redlands, CA, USA) and the Geospatial Modeling Environment software (Beyer, 2012) with the Cropland Data Layer (CDL, USDA, NASS 2012). As a metric for landscape complexity, we used the proportion of annual crop monocultures (0.16–0.77; cropland hereafter) as it represents a habitat that is frequented by lady beetles but is intrinsically species poor and, in contrast to seminatural habitat, is easy to unambiguously categorize. The proportion of cropland and seminatural habitat were negatively correlated (Pearson's *r *=* *−.88, *p *<* *.001) and the later produced essentially the same results when used in the analysis instead.

We sampled each field multiple times by sweep netting or hand collection from July through mid‐August. During this time, soybean aphid (*A. glycines*) populations usually reach high densities, but in 2012, they remained exceptionally low likely due to the severe drought in the Midwest (Liere et al., 2015). It was also difficult to find lady beetles (compared to our previous experience), and we succeeded in only eight soy fields and nine prairies. In total, we collected 243 beetles (*n* = 139 in prairie, *n* = 104 in soy) belonging to six aphidophagous species (Coccinellidae: Coccinellinae: Coccinellini) including the exotic *Coccinella septempunctata* L. (*n* = 49), *Harmonia axyridis* Pallas (*n* = 72), and *Hippodamia vairiegata* Goeze (*n* = 59), and the native *Cycloneda munda* Say (*n* = 16), *Hippodamia convergens* Guérin‐Méneville (*n* = 25), and *Hippodamia parenthesis* Dejean (*n* = 22; Gardiner et al., 2009). Collected beetles were immediately placed separately into microtubes containing 70% ethanol, transported to the laboratory on ice and preserved at −20°C until later analysis.

### 2.3 Sample processing

#### 2.3.1 Gut dissections

For both the beetles from the laboratory experiment and field collected specimens, the analysis of gut bacteria was conducted on dissected alimentary tracts. The beetles were carefully opened ventrally with sterilized fine‐tipped forceps in individual Petri dishes. Complete guts were isolated and stored in new 1.5‐ml microtubes containing 70% ethanol at −20°C. The ethanol was removed before DNA extraction with the PowerSoil Kit (MoBio Laboratories, Carlsbad, USA).

#### 2.3.2 Analysis of gut bacteria

We characterized the total gut bacterial community of lady beetles with Automated Ribosomal Intergenic Spacer Analysis (ARISA), a cost‐ and time‐efficient fingerprinting technique. ARISA detects bacterial phylotypes based on the length heterogeneity of the intergenic spacer region between the 16S and 23S rRNA genes (Fisher & Triplett, 1999). ARISA‐PCR was performed with 1406f/23Sr (Borneman & Triplett, 1997), a bacteria‐specific primer set with high taxonomic coverage (Purahong et al., 2015), as previously described (Shade et al., 2007; Yannarell, Kent, Lauster, Kratz, & Triplett, 2003).

We analyzed up to four technical PCR replicates for each sample of the feeding experiment due to the low number of biological replications. No technical replications were used for wild populations. Reagent‐only controls were included from the PCR step onwards. The PCR fragments were separated with a capillary sequencer (ABI 3730 DNA Analyzer, Applied Biosystems, Foster City, USA). The fragment sizes were determined by comparison with a custom internal 100–2,000 bp ROX‐labeled standard (BioVentures, Murfreesboro, USA) using GeneMarker v 1.5 (Soft Genetics LLC, State College, USA). Fragments were binned into operational taxonomic units (OTUs). The bin size was expanded from 1 bp for small fragments (200–550 bp) to 2 bp (551–700 bp), 3 bp (701–950 bp) and 5 bp for large fragments (951–1,200 bp) to account for the decreasing resolution with increasing fragment size (Abdo et al., 2006). Peaks that resulted from fluorescently labeled fragments were distinguished from the background noise by a custom R script (R Development Core Team, 2012) developed by Jones and McMahon (2009) based on Abdo et al. (2006).

Operational taxonomic units were treated as distinct bacterial taxa, and their relative fluorescence intensity was used as a proxy for relative taxon abundance within a sample to compare bacterial diversity and community structure between samples. ARISA can fail to accurately separate bacterial taxa at species level when multiple species have the same sequence length of the intergenic spacer and the method tends to underestimate diversity when species richness is high. Despite these limitations, other studies have demonstrated that patterns detected with ARISA are similar to those observed with sequencing‐based analysis at a fraction of the cost (van Dorst et al., 2014; Jami, Shterzer, & Mizrahi, 2014).

#### 2.3.3 Estimation of body fat content

We visually estimated the fat content in individual beetles during gut dissections. Beetles were assigned to the categories low, medium, and high fat content (Anderson, 1981): “Low”: little visual fat, mainly accumulated in the parietal layer; “Medium”, clearly visible fat accumulations also in regions of the gut or reproductive organs; “High”: fat filling and expanding the abdomen. Compared to whole body fat extraction, visual estimates of body fat do not provide quantitative data but allowed us to distinguish between storage fat and accumulated lipids in reproductive organs. Considering the fluctuations in total body fat in females during egg laying, estimates of storage fat provide a suitable assessment of the nutritional state.

### 2.4 Statistical analyses

All statistical analyses were performed in R (version 3.3.1, R Development Core Team, 2016) and R‐Studio (version 0.99.903, RStudio Team, 2015; Data files and R scripts in Appendices S2, S3, and S4 ). Means are reported ±1 *SD*.

### 2.5 Feeding experiment

For the feeding experiment, technical replications existed for all but three samples and were averaged prior to the analysis. The relationship between bacterial richness and the number of prey species in the meal (zero in the control, 1‐species diet, 5‐species diet) was analyzed with linear regression. The number of bacterial taxa in a sample was log‐transformed, and the model included number of technical replicates per sample as known prior weights, giving more weight to samples with more replications.

We analyzed the gut bacterial community assemblage using bacterial taxon relative abundances and calculating Bray–Curtis similarities (*vegan: vegdist*; Oksanen et al., 2017). We tested the effects of meal type (control, 1‐species diet, 5‐species diet) on community composition with permutational multivariate analysis of variance (perMANOVA; *adonis*; Oksanen et al., 2017) and permutation tests for the between group homogeneity in multivariate dispersions (*vegan: betadisper*,* permutest*; Oksanen et al., 2017; Anderson, 2006; McArdle & Anderson, 2001). Similarities between samples were visualized by NMDS (*metaMDS*; Oksanen et al., 2017).

### 2.4.2 | Field study

#### 2.4.2 | .1 Bacterial richness

We tested the effects of host‐specific factors, sex, field type, and proportion of annual cropland in the surrounding 2 km on the log‐transformed gut bacterial richness using linear mixed‐effects models (*nlme: lme;* Pinheiro & Bates, 2000). Alternative distributions for count data (Poisson, negative binomial) had higher AICc values (Akaike's information criterion corrected for small sample size; *stepAICc* function, *MASS* package, corrected for small sample sizes by C. Scherber, 2009, http://www.christoph-scherber.de/stepAICc.txt), and we therefore decided for a log‐transformation of the response. For the host‐specific factors, we constructed a custom contrast matrix that compared the six species according to three different attributes: origin (exotic vs. native), size (small vs. large), and genus (genus *Hippodamia* vs. non‐*Hippodamia*; Table 1). Models further included sex within species within collection site as a random effect. Variance heterogeneity between species was accounted for by introducing a variance function with different variances estimated for each species. Models were simplified based on AICc, starting with a model including the three‐way interaction. For the reported output, parameters were estimated based on restricted maximum likelihood (REML).

**Table 1:** Custom contrast matrix for lady beetle species

Lady beetle species	Genus group	Origin	Body size
*Coccinella septempunctata*	non‐*Hippodamia*	Exotic	Big
*Cycloneda munda*	non‐*Hippodamia*	Native	Small
*Harmonia axyridis*	non‐*Hippodamia*	Exotic	Big
*Hippodamia convergens*	*Hippodamia*	Native	Big
*Hippodamia variegata*	*Hippodamia*	Exotic	Small
*Hippodamia parenthesis*	*Hippodamia*	Native	Small

Small versus large body size refers to average measures of species elytron length (small <4.0 mm vs. big >4.5 mm; Julia Tiede (JT) & Claudio Gratton (CG), unpublished data).

#### 2.4.2 | .2 Bacterial community structure

Bacterial community composition in wild collected species was visualized as in the laboratory experiment with NMDS based on Bray–Curtis distances and by mean relative abundance of bacterial taxa per beetle species and habitat type (Fig. S2 in Appendix S1). We tested the effect of species, and species grouped by genus, origin, and body size on bacterial composition using separate (one‐way) perMANOVA (*adonis*; Oksanen et al., 2017). Species, as the best predictor, was included in a model testing the interactions between species and field type, and species and proportion cropland. Additionally, we tested the interaction between species and sex. All models included sex within species within collection site as random effect. Homogeneity of sample dispersion was tested (*vegan*:* betadisper*,* permutest*; Oksanen et al., 2017).

#### 2.4.2 | .3 Body fat content

We analyzed the proportion of beetles in three ordinal categories (low, medium, and high fat content) using cumulative link mixed‐effects models (*ordinal: CLMM*; Christensen, 2015) as a function of beetle species contrasts, field type, proportion cropland, and bacterial richness as fixed effects and beetle species within collection site as random effects. The full models included all two‐way interactions, and models were simplified as described above. To assess the effect of sex, three‐way interactions with sex were included in the best fit model and deleted from maximal models based on AICc.

## 3 RESULTS

### 3.1 Feeding experiment

**Figure 2:**
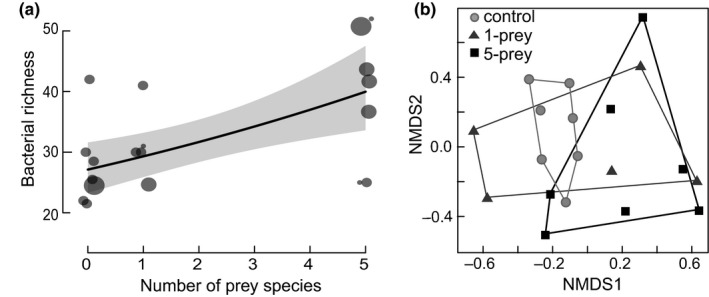
Bacterial (OTU) community richness and composition in feeding experiments. (a) Bacterial richness in guts of *C. maculata* as a function of the number of prey species in the meal (zero in the control, 1‐species diet, 5‐species diet). Points represent individual beetles and are scaled based on the number of averaged technical replicates, the black line and gray area show the predictions and 95% confidence interval of the linear regression model, respectively. (b) Community composition of bacteria in guts of *C. maculata* shown as NMDS (2D, stress = 0.19) based on Bray–Curtis dissimilarities of the relative abundance of bacterial taxa. Symbols represent individual beetles; colors and enclosing polygons refer to meal types.

In guts of the 19 beetles from the feeding experiment, we found 313 bacterial phylotypes (OTUs). The bacterial richness in individual beetle guts increased with the number of prey species in the meal (Table 2; Figure 2a) from 28 ± 7 (mean ± *SD*) in the beetles in the unfed (control) diet, to 31 ± 5 in the 1‐species diet, and 39 ± 11 in the 5‐species diet. Overall, we detected a significant but weak effect of the meal type on the bacterial community (perMANOVA; Table 2A; Figure 2b). In pairwise tests (Table 2b–d), the gut communities between beetles from the 1‐species diet and the 5‐species diet differed from the control but not from each other. Nonsignificant differences in sample dispersion (Table 2) indicated that the effects were driven by differences in the group centroids.

**Table 2:** Laboratory experiment results on the effect of meal type on gut bacteria in the gut of *C. maculata*

Linear model	*df*	Estimate ± *SE*	*t* value	*p* value
(Intercept)	1	3.301 ± 0.07	45.61	**<2e−16**
Number of prey species	1	0.078 ± 0.02	3.41	**.003**
Residuals	17			

perMANOVA	*df*	SS	*F* value	*p* value
(a) All meal types	2	1.13	1.74	**.014**
Residuals	16	5.20		[*R* ^2^ = 0.18]
(b) Control versus 1‐species diet	1	0.67	2.13	**.003**
Residuals	10	3.13		[*R* ^2^ = 0.18]
(c) Control versus 5‐species diet	1	0.67	2.12	**.008**
Residuals	12	3.79		[*R* ^2^ = 0.15]
(d) 1‐species diet versus 5‐species diet	1	0.35	1.00	.393
Residuals	10	3.49		[*R* ^2^ = 0.09]

PERMDISP	*df*	SS	*F* value	*p* value
Meal type	2	0.01	0.43	.659
Residuals	16	0.14		

Dark grey horizontal lines separate the different analysis. Linear model parameter estimates and standard errors on the effect of meal type on log‐transformed bacterial richness. PerMANOVA results on the effect of meal type on gut bacterial community in multiple (a) and pairwise contrasts (b–d). PERMDISP results on homogeneity of multivariate sample dispersion. *p* values <.05 are reported in bold numbers.

*df*, degrees of freedom; *SE*, standard errors; SS, sums of squares.

### 3.2 Field study

#### 3.2.1 Bacterial richness

**Figure 3:**
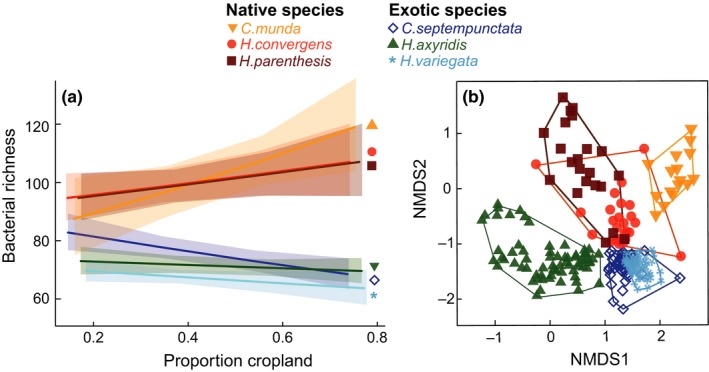
Bacterial (OTU) community richness and composition in wild beetle populations. (a) Effect of the interaction of beetle species and proportion cropland on the log‐transformed bacterial taxon richness (back‐transformed for illustrative purposes). Lines and shaded regions show response predictions and 95% confidence intervals from the mixed‐effects model. (b) Community composition of bacteria in gut samples of six wild populations of lady beetles visualized as NMDS (2D, stress = 0.20) based on Bray–Curtis dissimilarities of the relative abundance of bacterial taxa. Symbols and enclosing polygons represent individuals of different beetle species

In total, we found 551 bacterial taxa (OTUs) in the guts of 243 field collected beetles; the mean bacterial richness was 80 ± 20. Most of the variance in richness was explained by the differences between beetle species, which was higher in the three exotic species than in the three native species (Table 3 and Figure 3a; Table S3 in Appendix S1). Moreover, exotic and native species responded differently to landscape context: the bacterial richness in native species guts increased with increasing proportion of cropland surrounding the collection side, but decreased for exotic species (Tables 3 and Figure 3a; Table S3 in Appendix S1). Further, there was an effect of field type with higher bacterial richness in beetles collected in soy than in prairies (Table 3; Table S3 in Appendix S1). Sex had no effect.

**Table 3:** Field study results on gut bacteria and fat content of wild populations of lady beetles

Linear mixed model*	*df*	*denom. df*	χ^2^	*p* value
Species	3	31	177.55	**<.001**
Field type	1	14	12.22	**<.001**
Proportion crop	1	14	3.04	*.081*
Species × proportion crop	3	31	13.27	**.004**

perMANOVA	*df*	SS	*F* value	*p* value
(a) Species	5	27.51	26.89	**.001**
Residuals	237	48.49		[*R* ^2^ = 0.36]
(b) Origin	1	5.54	18.95	**.001**
Residuals	241	70.46		[*R* ^2^ = 0.07]
(c) Genus	1	5.39	18.39	**.001**
Residuals	241	70.61		[*R* ^2^ = 0.07]
(d) Size	1	5.14	17.48	**.001**
Residuals	241	70.86		[*R* ^2^ < 0.02]
(e) Sex	1	0.31	1.53	.148
Species × sex	5	1.11	1.08	.413
Residuals	231	47.1		[*R* ^2^ = 0.38]
(f) Field type	1	0.56	2.85	1.000
Species × field type	4	1.38	1.78	.147
Proportion crop	1	0.32	1.66	.722
Species × proportion crop	5	1.25	1.30	.485
Residuals	226	44.04		[*R* ^2^ = 0.42]

PERMDISP	*df*	SSqs	*F*‐value	*p*‐value
Species	5	1.39	39.02	**<.001**
Residuals	237	1.69		

Cumulative link mixed model**	*df*	*denom. df*	χ^2^	*p*‐value
Bacterial richness (log)	1	153	0.51	.476
Species	3	34	12.04	**.007**
Field type	1	13	4.33	**.037**
Proportion cropland	1	13	0.1	.753
Bacterial richness (log) × species	3	153	10.32	**.016**
Bacterial richness (log) × proportion crop	1	153	4.20	**.043**
Field type × proportion crop	1	13	2.97	*.085*

Dark grey horizontal lines separate the different analysis. Wald chi‐square tests from linear mixed model on the effect of species contrasts (native vs. exotic origin, small vs. big size; *Hippodamia* vs. other genera), sex, field type, and proportion cropland on log‐transformed bacterial richness. PerMANOVA results on the effects of species (a) and species grouped by origin, and size, (b–d), and sex (e), field type and proportion cropland after accounting for the effect of species and their interactions with species (f) on the bacterial community. PERMDISP results on homogeneity of multivariate sample dispersion. Likelihood‐ratio tests from cumulative link mixed model results on the effect of beetle species contrasts, log‐transformed bacterial richness, field type, and proportion cropland on beetle fat content. *p* values <.05 are reported in bold numbers and *p *<.10 in italics. Details on parameter estimates and standard errors are reported in Table S3 and S4 in Appendix S1.

*Mixed effects model denom. df = 159.

** Cumulative link mixed model denom. df = 153.

*df*, degrees of freedom; *denom. df*, denominator degrees of freedom; *SE*, standard errors; SS, sums of squares.

#### 3.2.2 Bacterial community structure

The bacterial assemblages were largely associated with beetle species identity (perMANOVA; Table 3a and Figure 3b). Origin, genus, and body size, also, had significant effects on the community structure, but the fit of the models was weaker (Table 3b–d). Sex, field type (corn vs. soy), and proportion cropland did not explain additional variability (Table 3e,f). The detected effects on the bacterial community might be partly driven by variances in sample dispersion between species (Table 3), but species also had distinct sets of abundant bacteria indicating compositional differences among species (Fig. S2 in Appendix S1).

#### 3.2.3 Body fat content

**Figure 4:**
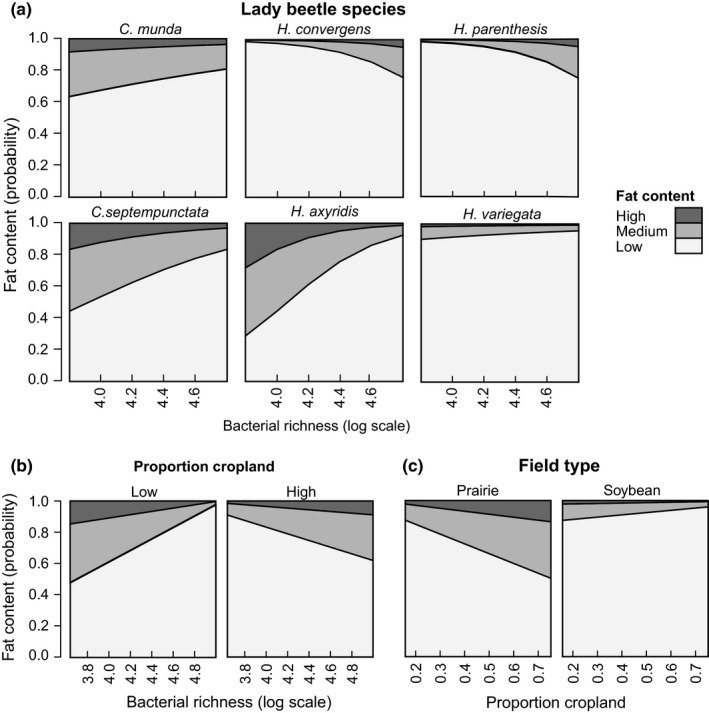
Body fat content in wild beetle populations. (a) Effects of the interactions of beetle species and log‐transformed gut bacterial taxon richness (OTUs), (b) proportion cropland and log‐transformed gut bacterial taxon richness (cropland was a continuous variable in the model but is shown as low and high for illustrative purposes), and (c) field type and proportion cropland on the proportion of beetles with low, medium, or high body fat as predicted by a cumulative link mixed model

The relative fat content of beetles was associated with species identity (Tables 3 and Table S4 in Appendix S1). Most beetles of the genus *Hippodamia* contained low body fat. Fat content of the two native *Hippodamia* species, *H. convergens* and *H. parenthesis,* increased with their gut bacterial richness, but this pattern was not observed in the exotic *H. parenthesis*. Conversely, in the exotic *C. septempunctata* and *H. axyridis*, beetles with a low gut bacterial richness were fattest (Table 3 and Figure 4a; Table S4 in Appendix S1). Gut bacterial richness also interacted with the proportion of cropland to affect variation in beetle fat content. Bacterial richness had a negative effect on fat content when the proportion of cropland was low and a positive effect when the beetles were collected in crop‐dominated areas (Tables 3 and Figure 4b; Table S4 in Appendix S1). Further, beetles collected in prairie had a higher fat content compared to soy (Table 3; Table S4 in Appendix S1) and tended to be fatter when the prairie was surrounded by cropland, but this interaction was only marginally significant (Table 3 and Figure 4c; Table S4 in Appendix S1). When sex was included as a fixed effect in the analysis, the interaction between prairie and the proportion of cropland also became significant. Additionally, we found an interaction between crop and sex with only females responding positive to increasing proportions of cropland. Further, there was an interaction between species and sex (Table S5 and S6 in Appendix S1).

## 4 DISCUSSION

We hypothesized that the diversity and composition of gut microbes in mobile arthropod predators would be affected by landscape context, both at the local (field) and at broader (among field, landscape) scale. Consistent with this prediction, we found that changes in landscape composition were associated with changes in richness of bacterial OTUs in the guts of beetles, but this effect was strongly species‐dependent. In fact, one of the strongest patterns observed in this study was the distinct difference in abundance and composition of gut bacteria across species of lady beetles. Moreover, a significant amount of bacterial community variation, and the response of microbes to landscape composition, was related to whether species were native or exotic, an unexpected finding. Native lady beetles had a richer gut bacterial community, and this richness increased as the landscape became more crop‐dominated; in contrast, the gut bacterial richness of exotic beetles was generally lower than that of natives and decreased as the amount of cropland increased in the landscape.

### 4.1 Species effects on bacterial richness and composition

The significant effect of lady beetle species on the gut bacterial community composition raises three nonmutually exclusive hypotheses about drivers of the composition the gut microbiome. That conspecific beetles had similar gut communities, even if they were sampled in different field types at distant collection sites, suggest that there may be a core group of species‐specific bacteria. Lady beetles are frequently infected with male‐killer bacteria (Majerus & Hurst, 1997; Weinert, Tinsley, Temperley, & Jiggins, 2007) but specific associations with gut microbes are largely unexplored, as is the case for most predatory insects. Shotgun‐sequencing of gut contents of lady beetles revealed potential symbionts (Paula et al., 2016). However, facultative gut symbionts were also detected in omnivorous ground beetles (Lundgren, Lehman, & Chee‐Sanford, 2007) and distinct gut communities in predatory ants (Anderson et al., 2012) and wasps (Mrázek, Strosová, Fliegerová, Kott, & Kopecný, 2008).

Another potential explanation is that species‐specific chemo‐physical characteristics of the gut select for colonization by certain bacteria (Dillon & Dillon, 2004; Nelson, Rogers, Carlini, & Brown, 2012). However, if this was a strong influencing factor, then we would expect that shared evolutionary history of beetles would result in the gut bacterial communities of closely related species to be more similar than distantly related species (Sanders et al., 2014). However, this was not the case for the three species of the genus *Hippodamia* in our study which had distinct bacterial assemblages more associated with whether they were exotic or native to the Midwestern USA. Although this study was not specifically designed to test for systematic differences in bacterial communities as a function of evolutionary relatedness or their exotic vs. native status, the patterns found in the most widespread beetle species in this area were strong and warrant additional study.

A third explanation for our findings of species‐specific differences in gut bacteria relates to differences in their diets, which could result in different sets of prey‐related bacteria. The laboratory experiment demonstrated that beetle gut communities could change relatively rapidly even within one species. Similar to our findings, *H. axyridis* gut microbes were enriched by aphid symbionts shortly after aphid ingestion (Paula et al., 2015). This hypothesis is further supported by a study on fruit fly species with distinct feeding habits, whose gut communities were different in wild populations but became similar on the same diet under laboratory conditions (Chandler et al., 2011). Thus, it is likely that at least some of the bacterial variation between lady beetle species was due to dietary differences maybe as a result of resource partitioning through differences in the dietary breadth, prey preferences, the ability to locate prey, preferred areas on a plant to forage, and the likelihood of switching habitats (Forbes & Gratton, 2011; Hodek & Honěk, 1996; Iperti, 1999; Schellhorn & Andow, 2005; Sloggett & Majerus, 2000). Studies that simultaneously identify food remains and microbes in gut contents (Paula et al., 2015; Tiede et al., 2016) could further illuminate the relation between diet and the gut microbiome.

### 4.2 Landscape effects on bacterial richness and composition

Other studies have shown that exotic species often dominate lady beetle communities in arable land. In this region, native species are mainly found in perennial grasslands and other seminatural habitats (Gardiner et al. 2009; Diepenbrock & Finke 2013; Grez et al. 2013). A similar pattern was found for native and exotic spider communities. An increasing amount of arable land is often associated with seminatural habitat fragmentation and more distant remnant patches are expected to harbor more dissimilar communities than close ones (Tscharntke et al., 2012). Thus, native beetles might have sampled a greater beta diversity of microbes from isolated natural habitat patches when located in landscapes with a high proportion of cropland. The preference of exotic beetles for homogenous agricultural habitats (i.e., crops fields) could have led to a reduced exposure to bacteria in the environment and therefore a lower gut bacterial richness. Additionally, a higher pathogen load in agricultural landscapes combined with higher antimicrobial defense in exotic species could contribute to the pattern of increasing microbial richness with increasing amount of cropland in native but not exotic lady beetles. Along these lines, farmland frogs harbored more potentially harmful bacteria in their guts than frogs from natural habitats (Chang, Huang, Lin, Huang, & Liao, 2016). A strong antimicrobial defense has been detected in the exotic *H. axyridis* (Beckert et al., 2015; Gross, Eben, Müller, & Wensing, 2010; Vilcinskas, Mukherjee, & Vogel, 2013) and is suggested as a potential mechanism driving invasive predator success (enemy release hypothesis; Roy, Handley, Schönrogge, Poland, & Purse, 2011).

The specific habitat type in which beetles were collected, soy compared to prairie, was another strong predictor for bacterial richness. In contrast, to our expectation that beetles from prairie would have a richer gut community, we found more bacterial diversity in the guts of beetles from soy. This finding could be partly attributed to a drought that affected the soybean plants and aphid populations in southern Wisconsin (Mallya, Zhao, Song, Niyogi, & Govindaraju, 2013). The low availability of soybean aphids, the principal prey of lady beetles in this crop, likely increased the consumption of alternative prey (Iperti, 1999; Sloggett & Majerus, 2000). A broader diet in soybean would expose the beetles to a greater variability of environmental bacteria compared to a diet of mainly aphids. In *H. axyridis*, aphid–symbionts were detected up to 96 hr after aphid consumption (Paula et al., 2015). Prairie plant communities were more resilient to the drought than row crops (Joo et al., 2016) and likely allowed the aphidophagous lady beetles in our study to be more selective in their prey choice.

Additionally, differences in local food availability between the two habitat types could have led to differences in residency time. The beetles we collected in soybean might have switched from another (crop‐) habitat not long before (Forbes & Gratton, 2011) and carried over bacteria and higher food availability in prairie could have increased small‐scale foraging. The lack of information on how much time a beetle has spent in the field where it was sampled may to some degree confound the local and the landscape scale used in our study.

Studies that compare samples from multiple seasons and years could help to further elucidate what shapes the gut community. Our results indicate that the total gut community of lady beetles can be divided into a stable and a variable part. The core OTUs that form similar gut communities in conspecific beetles collected from different habitats and at distant collection sites are likely also relatively stable between seasons and years. More transient, food‐related bacterial taxa should be highly variable and respond to annual and seasonal changes in food availability, and the variations might be more extreme in crop‐dominated regions with many ephemeral food sources. For example, in a year with high aphid abundance in soy we would expect the pattern we found to be reversed, with lower bacterial richness found in beetles from soy as compared to beetles that forage in prairies.

### 4.3 Microbe and landscape effects on ladybeetle fat content

We posit that the higher gut bacterial richness in beetles from soy fields compared to prairies is an indicator of consumption of mixed alternative resources in absence of soybean aphids. This interpretation is consistent with the findings that beetles collected in prairie had a higher fat content compared to soy‐collected beetles, indicating superior feeding conditions and a better outcome for body condition in prairie compared to aphid–depauperate soy. Landscape context on a broad scale had no effect itself but mediated the effect of bacterial richness on body fat of beetles: As bacterial richness increased, beetles became fatter in agriculturally dominated landscapes, while for beetles collected in landscapes with few crops, higher bacterial richness was associated with lower fat content. Generalist predators can benefit from some proportion of cropland, which periodically provides abundant food resources (Rand & Tscharntke, 2007) but may benefit more from the inclusion of alternative resource with complementary nutrients in simplified landscapes in which they mainly find crop pests. Other studies on predatory beetle body condition found positive effects of landscape heterogeneity (Östman et al., 2001) and succession‐related food supply and diversity of wildflower habitats (Barone & Frank, 2003).

Although landscape context clearly had an impact on gut microbiota, and landscape context and gut microbial richness together affected the fat content of lady beetles, the ultimate causal mechanisms remain to be explored. We propose that food resource abundance and diversity in the local habitat could be one of the main drivers for both gut bacterial richness and host fat content. Further, diet‐related bacteria can potentially affect host fitness directly when they serve as a supplemental food source, temporarily contribute to digestion processes (Bouchon et al., 2016) or facilitate adaption to novel food sources (Chu, Spencer, Curzi, Zavala, & Seufferheld, 2013). However, if and to what extend a predator benefits from a mixed diet (Evans, Stevenson, & Richards, 1999; Harwood et al., 2009; Lefcheck, Whalen, Davenport, Stone, & Duffy, 2012; Lundgren, 2009) and diverse gut bacteria depends on host species: In our study, the two native beetles *H. convergens* and *H. parenthesis* had more body fat when their guts harbored many different bacterial. In contrast, the exotic *C. septempunctata* and *H. axyridis* were fatter when their gut bacterial communities were species poor. This finding might reflect that exotic species are better adapted to homogenous conditions in cropland than native species and therefore often dominate coccinellid communities in cultivated habitats (Bahlai, Colunga‐Garcia, Gage, & Landis, 2013).

## 5 CONCLUSION

A key finding of this study is that mobile predatory insects have a species‐specific set of gut bacteria that is stable over a range of environmental conditions. However, landscape and habitat‐associated differences in where they are collected can alter this base assemblage. Although the mechanisms for these patterns are not resolved, the strong differences between exotic and native species and the contrasting effects of landscape context on gut bacteria suggest inherent differences in habitat and prey use among these groups. Moreover, that landscape context can also affect host performance as indicated by fat content, both directly and indirectly via gut microbiota, potentially indicates a novel mechanism through which human‐altered landscapes can affect invertebrate predators. The method we used to analyze gut bacterial communities allowed us to rapidly compare samples from multiple species and locations but does not provide information on taxon identity. Sequencing‐based technologies in combination with reference databases for taxon identification are an ideal next step. This could help identify the core microbes of different species, their relationship to the host and response to environmental factors. We focused on bacterial microbes which are thought to comprise the greatest fraction of organisms in the guts of many insect (Engel & Moran, 2013), but further studies could expand the range to other potential interaction partners, like fungi, protists, and archaea. Overall, our study illustrates the importance of both resource and landscape‐based influences on gut microbiota and their interactions with species‐specific traits including foraging behavior and physiology.

## DATA ACCESSIBILITY

The data and R scripts used for data analysis are provided in the in Appendices S2, S3, and S4.

## AUTHOR CONTRIBUTIONS

JT, CG, and KDM conceived and designed the study; JT performed the laboratory experiments and collected field samples; JT and JM performed molecular analysis and processed the data; JT, CG, and CS analyzed output data. JT wrote the first draft of the manuscript, and all authors were substantially involved in discussions and editing.

## CONFLICT OF INTEREST

None declared.
