# Supplementary material for: Gut microbiomes of mobile predators vary with landscape context and species identity
Source: Ecol Evol. 2017 Sep 12;7(20):8545–57. doi: 10.1002/ece3.3390 (PMC5648672; doi:10.1002/ece3.3390)
Supplement: Supplementary file 1 [file ECE3-7-8545-s001.docx]

**Appendix S1: Supplementary figures and tables S1 – S6**

**Manuscript accepted for publication in Ecology & Evolution on 2017/07/26**

[**https://doi.org/10.1002/ece3.3390**](https://doi.org/10.1002/ece3.3390)

**Title: “Gut microbiomes of mobile predators vary with landscape context and species identity”**

**Authors:** Julia Tiede^1,2,4*^, Christoph Scherber^1,2^, James Mutschler^3^, Katherine D. McMahon^3^, Claudio Gratton^4^

^1^ Institute of Landscape Ecology, University of Muenster, Heisenbergstr. 2, 48149, Muenster, Germany

^2^ Department of Crop Sciences, University of Goettingen, Grisebachstr. 6, 37077, Goettingen, Germany

^3^ Departments of Civil and Environmental Engineering and Bacteriology, University of Wisconsin-Madison, 1415 Engineering Drive, Madison, Wisconsin, USA

^4^ Department of Entomology, University of Wisconsin-Madison, 1630 Linden Drive, Madison, WI, 53706, USA


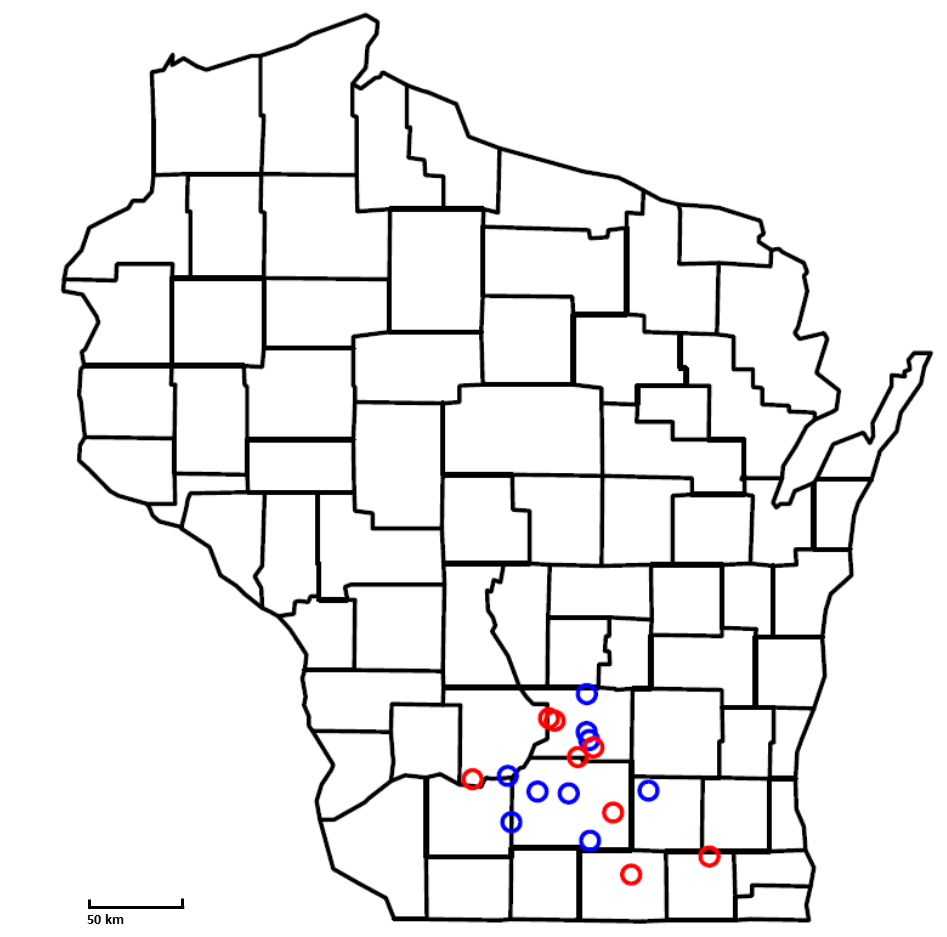


# Figure S1 Map of sampling locations.

Locations of soybean fields (red points) and prairies (blue points) around Madison, Wisconsin, USA, in which the lady beetle specimen included in our study were collected between July and August 2012.


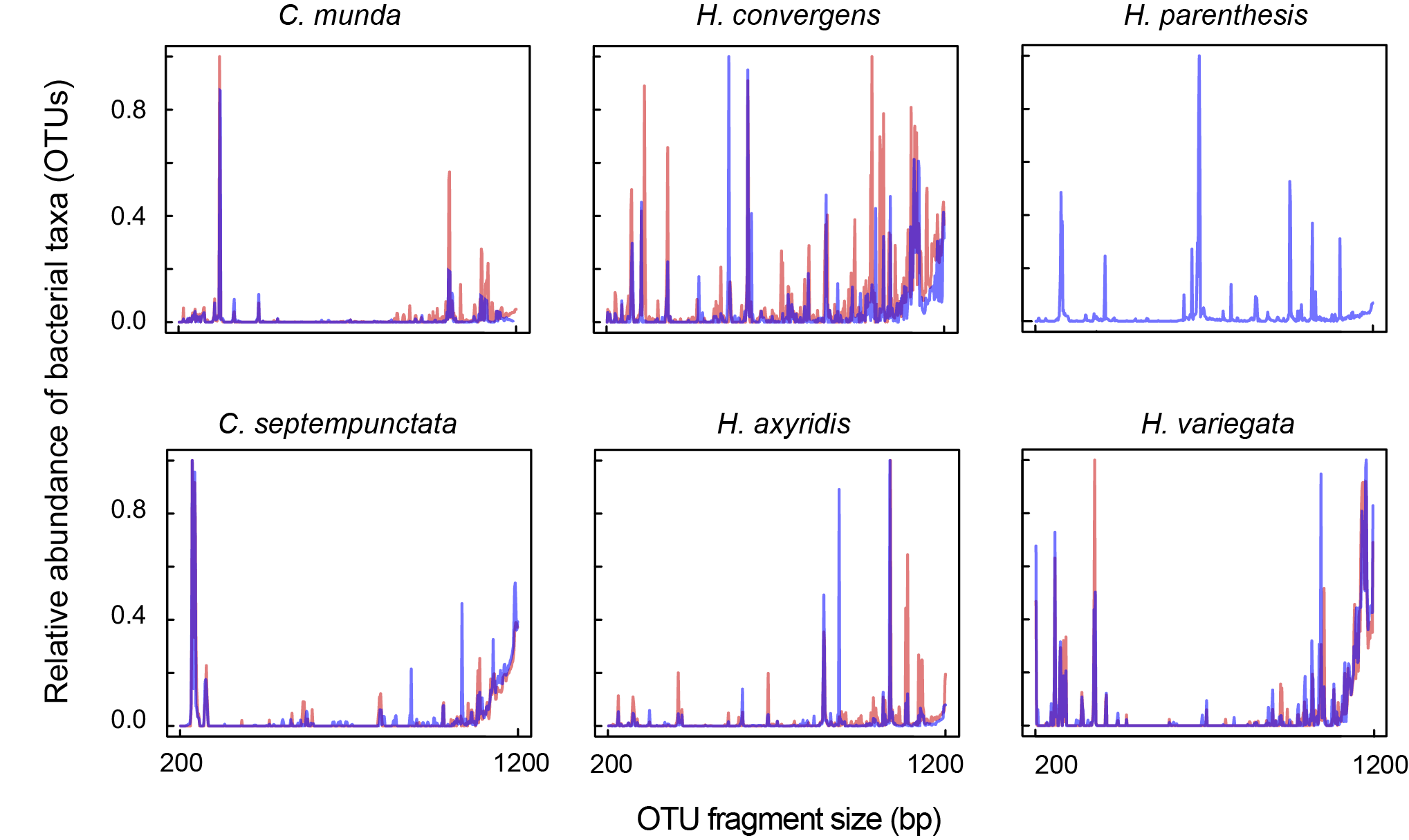


# Figure S2. Effect of beetle species and field type on relative abundance of bacterial taxa.

Mean relative abundance of bacterial taxa represented by operational taxonomic units (OTUs) of different size (base pairs) in different species of lady beetles. The blue and red lines represent the bacterial community in beetles from prairie and soy, respectively.

# Table S3. Field study results for effects on gut bacterial richness.

Parameter estimates and standard errors from the linear mixed model (corresponding to Table 2) on the effect of species contrasts (native vs exotic origin, small vs big size; *Hippodamia* vs other genera), field type, and proportion cropland in 2 km on log-transformed richness of gut bacterial taxa. The model included beetle gender within species within collection site as random effect (n = 243 beetles, n = 17 sites, n = 54 beetles within sites, n = 84 gender within species within sites). The estimated variances of the random effects were essentially 0, with a residual variance of (0.26)². Variance heterogeneity between species was accounted for by introducing a variance function with different variances estimated for each species (*C. munda* = 1, *H. convergens* = 1.053, *H. axyridis* = 1.002, *H. parenthesis* = 1.467, *C. septempunctata* = 0.903, and *H. variegata* = 0.778). *P*-values <0.05 are reported in bold numbers, while *P*<0.10 are in italics.

| Term | Value | SS | Df | *t-*value | *P*-value |
| --- | --- | --- | --- | --- | --- |
| (Intercept) | 4.369 | 0.035 | 159 | 124.609 | **<0.001** |
| Species-origin | -0.044 | 0.043 | 31 | -1.039 | 0.307 |
| Species-genus | 0.018 | 0.029 | 31 | 0.623 | 0.538 |
| Species-size | 0.084 | 0.032 | 31 | 2.579 | **0.015** |
| Field type-soy | 0.094 | 0.027 | 14 | 3.495 | **0.004** |
| Proportion crop | 0.064 | 0.080 | 14 | 0.803 | 0.435 |
| Species-origin x proportion crop | -0.304 | 0.088 | 31 | -3.469 | **0.002** |
| Species-genus x proportion crop | 0.036 | 0.064 | 31 | 0.566 | 0.576 |
| Species-size x proportion crop | -0.122 | 0.064 | 31 | -1.891 | *0.068* |

SS= Sums of squares

Df = Degrees of freedom

# Table S4. Field study results for effects on beetle fat content.

Parameter estimates and standard errors from the cumulative link mixed model (corresponding to Table 3) on the effect of beetle species contrasts, log-transformed bacterial richness, field type, and proportion cropland on fat content of lady beetles. The model included beetle gender within species within collection site as random effect (n = 242 beetles, n = 17 sites, n = 54 species within sites, n= 84 gender within beetles within sites). The estimated variances of the random effects were 0.601 for gender within species within site, 0 for species within site, and 0.510 for site. Model selection was based on stepwise deletion of predictors based on AICc. *P*-values <0.05 are reported in bold numbers, while *P*<0.10 are in italics.

| Term | Estimate | SE | z-value | *P*-value |
| --- | --- | --- | --- | --- |
| Low\|medium | -14.344 | 7.526 | -1.906 |  |
| Medium\|low | -12.122 | 7.497 | -1.617 |  |
| Bacterial richness (log) | -3.122 | 1.714 | -1.821 | *0.069* |
| Species-origin | 5.512 | 4.538 | 1.215 | 0.225 |
| Species-genus | 6.280 | 4.205 | 1.494 | 0.135 |
| Species-size | -3.778 | 4.512 | -0.837 | 0.402 |
| Field type-soy | 0.810 | 1.384 | 0.585 | 0.559 |
| Proportion crop | -28.426 | 15.685 | -1.812 | *0.070* |
| Bacterial richness (log) x Species-origin | -1.264 | 1.011 | -1.250 | 0.211 |
| Bacterial richness (log) x Species-genus | -1.232 | 0.971 | -1.268 | 0.205 |
| Bacterial richness (log) x Species-size | 0.895 | 1.027 | 0.872 | 0.383 |
| Bacterial richness (log) x Proportion crop | 7.322 | 3.628 | 2.018 | **0.044** |
| Proportion crop x Field type-soy | -5.413 | 2.940 | -1.841 | *0.066* |

SE = Standard errors

# Table S5. Field study results for effects on beetle fat content - Likelihood-ratio tests from clmm model including gender as fixed term.

Likelihood-ratio tests from cumulative link mixed model results on the effect of beetle species contrasts, log-transformed bacterial richness, field type, and proportion cropland on beetle fat content. *P*-values <0.05 are reported in bold numbers. Details on parameter estimates and standard errors reported in Table S6.

| Term | | Df |  | Chi² | *P*-value |
| --- | --- | --- | --- | --- | --- |
|  | Bacterial richness (log) | 1 |  | 0.58 | 0.448 |
|  | Species | 3 |  | 13.25 | **0.004** |
|  | Field type | 1 |  | 4.45 | **0.035** |
|  | Proportion cropland | 1 |  | 0.17 | 0.680 |
|  | Bacterial richness (log) x species | 3 |  | 12.78 | **0.005** |
|  | Bacterial richness (log) x proportion crop | 1 |  | 5.32 | **0.021** |
|  | Field type x proportion crop | 1 |  | 3.95 | **0.047** |
|  | Species x gender | 3 |  | 10.51 | **0.015** |
|  | Proportion cropland x gender | 1 |  | 5.18 | **0.023** |

Df = Degrees of freedom

SE = Standard errors

SS = Sums of squares

# Table S6. Field study results for effects on beetle fat content - Parameter estimates from clmm model including gender as fixed term.

Parameter estimates and standard errors from the cumulative link mixed model (corresponding to Table S5) on the effect of beetle species log-transformed bacterial richness, field type, and proportion cropland on fat content of lady beetles. The model included beetle species within collection site as random effect (n = 242 beetles, n = 17 sites, n = 54 species within sites, n= 84 gender within species within site). The estimated variances of the random effects were essentially 0. Model selection was based on stepwise deletion of predictors based on AICc. *P*-values <0.05 are reported in bold numbers, while *P*<0.10 are in italics.

| Term | Estimate | SE | z-value | *P*-value |
| --- | --- | --- | --- | --- |
| Low\|medium | -16.102 | 7.755 | -2.076 |  |
| Medium\|low | -13.802 | 7.724 | -1.787 |  |
| Bacterial richness (log) | -3.554 | 1.770 | -2.008 | **0.045** |
| Species-origin | 4.973 | 4.576 | 1.087 | 0.277 |
| Species-genus | 7.568 | 4.310 | 1.756 | *0.079* |
| Species-size | -4.829 | 4.550 | -1.061 | 0.288 |
| Field type-soy | 1.237 | 1.456 | 0.849 | 0.396 |
| Proportion crop | -32.140 | 16.360 | -1.965 | **0.049** |
| Bacterial richness (log) x Species-origin | -1.212 | 1.018 | -1.190 | 0.234 |
| Bacterial richness (log) x Species-genus | -1.595 | 0.997 | -1.599 | 0.110 |
| Bacterial richness (log) x Species-size | 1.069 | 1.036 | 1.032 | 0.302 |
| Bacterial richness (log) x Proportion crop | 8.568 | 3.802 | 2.254 | **0.024** |
| Proportion crop x Field type-soy | -6.581 | 3.088 | -2.131 | **0.033** |
| Proportion crop x Gender-male | -2.457 | 0.742 | -3.313 | **0.001** |
| Species-origin x Gender-male | 0.297 | 0.392 | 0.758 | 0.449 |
| Species-genus x Gender-male | 0.951 | 0.356 | 2.667 | **0.008** |
| Species-size x Gender-male | 0.642 | 0.364 | 1.763 | *0.078* |

SE = Standard errors
