# Supplementary material for: Gut microbiomes of mobile predators vary with landscape context and species identity
Source: Ecol Evol. 2017 Sep 12;7(20):8545–57. doi: 10.1002/ece3.3390 (PMC5648672; doi:10.1002/ece3.3390)
Supplement: Supplementary file 2 [file ECE3-7-8545-s002.docx]

**Appendix S2: R-Scripts used for data analysis**

**Manuscript accepted for publication in Ecology & Evolution on 2017/07/26**

[**https://doi.org/10.1002/ece3.3390**](https://doi.org/10.1002/ece3.3390)

**Title: “Gut microbiomes of mobile predators vary with landscape context and species identity”**

**Authors:** Julia Tiede^1,2,4*^, Christoph Scherber^1,2^, James Mutschler^3^, Katherine D. McMahon^3^, Claudio Gratton^4^

^1^ Institute of Landscape Ecology, University of Muenster, Heisenbergstr. 2, 48149, Muenster, Germany

^2^ Department of Crop Sciences, University of Goettingen, Grisebachstr. 6, 37077, Goettingen, Germany

^3^ Departments of Civil and Environmental Engineering and Bacteriology, University of Wisconsin-Madison, 1415 Engineering Drive, Madison, Wisconsin, USA

^4^ Department of Entomology, University of Wisconsin-Madison, 1630 Linden Drive, Madison, WI, 53706, USA

**##################################################################################**

**### 1. Lab feeding experiment with the lady beetle *Coleomegilla maculata***

# read datafile with individual samples

diet.experiment1=read.table("Tiede et al_Appendix S3_Data_lab-experiment.txt",h=T,sep="\t")

str(diet.experiment1)

names(diet.experiment1)

# count how often a sample was sequenced

library(plyr)

count=count(diet.experiment1,c("ID_sample"))

diet.experiment2=merge(count,diet.experiment1,by=c("ID_sample"))

names(diet.experiment2)

# aggregate samples that have been sequences multiple times by sample ID ("ID_sample")

diet.experiment=aggregate(.~ID_sample+treatment+prey,

data=diet.experiment2[,c(1:4,6:319)], function(x)mean(x))

str(diet.experiment)

names(diet.experiment)

**##################################################################################**

**## 1.1 lab experiment - gut bacterial richness**

# test effect if gut bacterial richness is affected by number of prey species ("prey") in the diet

# on log-transformed bacterial richness ("OTUs") in beetle guts (number of OTUs per sample technical-replicate averaged by sample)

# with lm with averaged sample replicates, weighted by the number of replicates ("freq")

lm1=lm(log(OTUs)~prey,diet.experiment,weights=freq)

plot(lm1)

summary(lm1)

anova(lm1)

#plot model effets

library(effects)

plot(allEffects(lm1,partial.residuals=T))

**##################################################################################**

**## 1.2 lab experiment - gut bacterial community composition**

# test effect of number of prey species ("prey") in the diet on bacterial on community composition in beetle guts

# Effect of prey on community composition?

names(diet.experiment)

com=diet.experiment[,6:318] #community data

env=diet.experiment[,1:5] #environmental variables

## make distance matrix

library(vegan)

dist<-vegdist(com, method="bray")

## Permutation test for homogeneity of multivariate dispersions

bet<-betadisper(dist, env$treatment)

pmod <- permutest(bet, pairwise = TRUE)

pmod

## perMANOVA

perm <- adonis(dist ~ treatment, data=env)

perm

## perMANOVA pairwise tests: control treatment vs 1-prey diet

com.control.vs.1prey=subset(com, env$treatment!="5-prey")

env.control.vs.1prey=subset(env, env$treatment!="5-prey")

dist.control.vs.1prey<-vegdist(com.control.vs.1prey, method="bray") #distance matrix

perm.control.vs.1prey <- adonis(dist.control.vs.1prey ~ treatment, data=env.control.vs.1prey)

perm.control.vs.1prey

# perMANOVA pairwise tests: control treatment vs 5-prey diet

com.control.vs.5prey=subset(com, env$treatment!="1-prey")

env.control.vs.5prey=subset(env, env$treatment!="1-prey")

dist.control.vs.5prey<-vegdist(com.control.vs.5prey, method="bray")

perm.control.vs.5prey <- adonis(dist.control.vs.5prey ~ treatment, data=env.control.vs.5prey)

perm.control.vs.5prey

# perMANOVA pairwise tests: 1-prey diet vs 5-prey diet

com.1prey.vs.5prey=subset(com, env$treatment!="control")

env.1prey.vs.5prey=subset(env, env$treatment!="control")

dist.1prey.vs.5prey<-vegdist(com.1prey.vs.5prey, method="bray")

perm.1prey.vs.5prey <- adonis(dist.1prey.vs.5prey ~ treatment, data=env.1prey.vs.5prey)

perm.1prey.vs.5prey

#plot nMDS

nMDS<-metaMDS(com, dist="bray", k=2)

plot(nMDS,type="n", display=c("sites"),main="Lab experiment")

points(nMDS, display=c("sites"), col=factor(env$treatment), pch=19, cex=1)

legend("topleft", bty="n", legend= levels(factor(env$treatment)), col=seq_along(factor(env$treatment)), pch=16, cex=0.9)

ordihull(nMDS, env$treatment, label=F) #draw polygon

**##################################################################################**

**# 2. Field populations of lady beetles**

#read data

gut <-read.table("Tiede et al_Appendix S4_Data_field-study.txt",sep="\t",head=T)

names(gut)

str(gut)

#make costum contrasts for origin, genus, and body size of lady beetle species

contrasts(gut$species)

levels(gut$species)

gut$species=ordered(gut$species,levels=c("C.mu","H.c","H.p","C.s","H.ax","H.v"))

gut$species2=gut$species

contrasts(gut$species2)

cmat=cbind(

c(-1,-1,-1,1,1,1), # native vs. exotic origin

c(1,-1,-1,1,1,-1), # genus Hippodamia vs non-Hippodamia

c(-1,1,1,1,-1,-1)) # small vs. large body size

dimnames(cmat)=list(levels(gut$species2),c("origin","genus","size"))

contrasts(gut$species2,how.many=3)=cmat

contrasts(gut$species2)

**##################################################################################**

**## 2.1 field populations - gut bacterial richness**

# test effect of number of species-contrasts ("species2"), field type ("habitat"; soy vs prairie)

# and proportion of annual crops in 2km radius of the collection site ("crop2.0")

# on log-transformed bacterial richness ("OTUs") in individual beetle guts

# with nlme

library(nlme)

#model without transformation

m0=lme(OTUs~species2*gender*habitat*crop2.0,random=~1|site/species2/gender,data=gut)

plot(m0)

# model with log transformation

m1=lme(log(OTUs)~species2*gender*habitat*crop2.0,random=~1|site/species2/gender,data=gut)

plot(m1) # residuals look better with log transformation

m1a=update(m1,weights=varIdent(form=~1|species)) #account for non-homogenous variance between species

m1b=update(m1,correlation=corCompSymm(form=~as.numeric(lat+lon))) #account for spatial autocorrelation

plot(ACF(m1,form=~as.numeric(lat+lon)),alpha=0.05)

library(MuMIn)

AICc(m1,m1a,m1b) #m1a is the best model!

m1e=update(m1a, method="ML")

library(MASS)

m1f=stepAICc(m1e)

m1g=update(m1f,method="REML")

library(car)

Anova(m1g, type="II")

summary(m1g)

mean(log(gut$OTUs)) #for interpretation of effect size

library(effects)

plot(allEffects(m1g, transformation=list(link="log",inverse="exp"),

partial.residuals=TRUE, multiline=TRUE))

#model m1g

m1g$call

#effects fit and SE

ee=Effect("habitat", mod=m1g,transformation=list(link=log,inverse=exp),se = TRUE)

as.data.frame(ee)

#prairie n= 139

#soy n=104

**##################################################################################**

**## 2.2 field populations - gut bacterial community composition**

#1) test if disperion is homogenous

#2) test effect of species, origin, genus, body size, and gender

#3) test the interaction between species and gender

#4) test the interaction between species and field type ("habitat"; soy vs prairie)

# and species and proportion of annual crops in 2km radius of the collection site ("crop2.0")

# on bacterial community composition in guts of lady beetles sampled from field populations

communities=gut

names(communities)

com=communities[,21:572] #community data

env=communities[,1:20] #environmental variables

# make distance matrix

library(vegan)

dist<-vegdist(com, method="bray")

#1) Permutation test for homogeneity of multivariate dispersions

bet<-betadisper(dist, env$species, type = "centroid", bias.adjust = TRUE)

pmod <- permutest(bet, pairwise = TRUE)

pmod

#2) PERMANOVA tests on single effects

#effect of beetle species

ado1<-adonis(dist~(env$species),strata= env$site)

#effect of species origin (native vs exotic)

ado2<-adonis(dist~(env$origin),strata= env$site)

ado1

#effect of genus (Hippodamia vs non-Hippodamia)

ado3<-adonis(dist~(env$genus),strata= env$site)

ado3

#effect of body size (small vs. big)

ado4<-adonis(dist~(env$size),strata= env$site)

ado4

#effect of gender (female vs. male)

ado5<-adonis(dist~(env$gender),strata= env$site)

ado5

#3) PERMANOVA test for interactions of species and gender

ado1a<-adonis(dist~(env$species*env$gender),strata= env$site)

ado1b<-adonis(dist~(env$gender*env$species),strata= env$site)

#4) PERMANOVA test for interactions of species and landscape factors

ado2a<-adonis(dist~(env$species*env$crop2.0+env$species*env$habitat),strata= env$site)

ado2a

ado2b<-adonis(dist~(env$species*env$habitat+env$species*env$crop2.0),strata= env$site)

ado2b

#plot effect of species

m1<-metaMDS(com, dist="bray", k=2) #nMDS

plot(m1,type="n", display=c("sites"),main="Effect of species")

points(m1, display=c("sites"), col=factor(env$species), pch=16, cex=1.2)

legend("topleft", bty="n", legend= levels(factor(env$species)), col=seq_along(factor(env$species)), pch=16, cex=0.9)

ordihull(m1, env$species, label=F, cex=0.8)

**##################################################################################**

**## 2.3 field populations - body fat**

# 1) test effect of number of species identity (the contrasts dont work here!),

# habitat type ("habitat"; soy vs prairie)

# and proportion of annual crops in 2km radius of the collection site ("crop2.0")

# on body fat ("fat") in individual beetles

# with clmm

# 2) redo the test with gender as fixed effect in the model

fat.content=subset(gut, sampleID!="SHx50")#remove sample with NA for fat content

library(ordinal)

fat.content$fat.ord=ordered(fat.content$fat)

plot(fat.ord~species2, fat.content)

levels(fat.content$fat)=c("low","medium","high")

fat.content$fat=ordered(fat.content$fat)

fat.content$fat

#log-transform bacterial richness

fat.content$LOTU=log(fat.content$OTUs)

#nullmodell

fat0=clmm(fat~ 1+(1|site/species2/gender),data=fat.content,Hess=TRUE)

#model with all 2-way interactions

fat1=clmm(fat~ (LOTU+crop2.0+species2+habitat)^2 +(1|site/species2/gender),data=fat.content,Hess=TRUE)

library(RVAideMemoire)

Anova(fat1, type="II") # mainly LOTU, habitat and species2 significant

#remove LOTU:habitat

fat2=update(fat1,~.-LOTU:habitat)

Anova(fat2,type="II")

#remove species:habitat

fat3=update(fat2,~.-species2:habitat)

Anova(fat3,type="II")

#remove crop2.0:species

fat4=update(fat3,~.-crop2.0:species2)

Anova(fat4,type="II")

#remove crop2.0:habitat

fat5=update(fat4,~.-crop2.0:habitat)

Anova(fat5,type="II")

AICc(fat0, fat1, fat2, fat3, fat4, fat5) # fat4 is the best model!

fat4$call

Anova(fat4,type="II")

summary(fat4)

plot(allEffects(fat4),style="stacked")

##################################################################################

**## 2.3 – version2 - field populations - body fat**

#test best model from first approach but include gender interactions

fat1c=clmm(formula = fat ~ LOTU + crop2.0 + species2 + habitat

+ LOTU:gender + crop2.0:gender + species2:gender + habitat:gender

+ LOTU:crop2.0 + LOTU:species2 + crop2.0:habitat

+ LOTU:crop2.0:gender + LOTU:species2:gender + crop2.0:habitat:gender

+ (1|site/species2/gender), data = fat.content, Hess = TRUE)

Anova(fat1c,type="II")

#remove crop2.0:habitat:gender

fat2c=update(fat1c,~.-crop2.0:habitat:gender)

Anova(fat2c,type="II")

#remove LOTU:crop2.0:gender

fat3c=update(fat2c,~.-LOTU:crop2.0:gender)

Anova(fat3c,type="II")

#remove LOTU:species2:gender

fat4c=update(fat3c,~.-LOTU:species2:gender)

Anova(fat4c,type="II")

#remove habitat:gender

fat5c=update(fat4c,~.-habitat:gender)

Anova(fat5c,type="II")

#remove LOTU:gender

fat6c=update(fat5c,~.-LOTU:gender)

Anova(fat6c,type="II")

AICc(fat0, fat4, fat1c, fat2c, fat3c, fat4c, fat5c, fat6c) # fat6c is the best model

fat6c$call

Anova(fat6c,type="II")

summary(fat6c)

plot(allEffects(fat6c),style="stacked")
